# Management of preexisting pelvic organ prolapse in pregnancy complicated with preterm premature rupture of membrane: a case report

**DOI:** 10.1186/s13256-023-03901-5

**Published:** 2023-06-09

**Authors:** Muhudin Arusi, Elham Abdulhakim, Yasin Awol, Hassen Mosa

**Affiliations:** 1 Department of Obstetrics and Gynecology, Worabe Comprehensive Specialized Hospital, Worabe, Ethiopia; 2 Department of Neurology, Worabe Comprehensive Specialized Hospital, Worabe, Ethiopia; 3 Department of Medical Laboratory, Worabe Comprehensive Specialized Hospital, Worabe, Ethiopia; 4 Department of Midwifery, College of Medicine and Health Sciences, Werabe University, Worabe, Ethiopia

**Keywords:** Pelvic organ prolapsed, Preterm premature rupture of membrane, Pessary, Cesarean hysterectomy

## Abstract

**Background:**

Pregnancy management is difficult when pelvic organ prolapse already exists. During pregnancy, childbirth, and the days following, clinicians may come across situations that present management dilemmas. Here, we present conservative management of preexisting pelvic organ prolapse in pregnancy complicated with preterm premature rupture of membrane up to term.

**Case presentation:**

A 35-year-old Ethiopian woman, gravida V, para IV, visited our emergency obstetrics and gynecology department at 32 weeks and 1 day of pregnancy in a prolapsed uterus on the 4th of April 2022. She was referred from primary hospital as a case of preterm pregnancy, pelvic organ prolapse, and preterm premature rupture of membrane after she presented with complaints of passage of clear liquor of 10 hours duration. She was successfully managed conservatively without application of pessary until she gave birth to a 3200 g healthy male neonate by elective cesarean section at 37 weeks of gestational age. At the same operation, cesarean hysterectomy was done.

**Conclusion:**

Women with preexisting pelvic organ prolapse complicated by premature rupture of membrane during the third trimester of pregnancy can be treated without the use of a pessary. Our case shows the importance of conservative management, which includes strict antenatal follow-ups, lifestyle modifications, and manual uterine reduction. Due to potential intrapartum problems from induction of labor with the occurrence of severe pelvic organ prolapse, we recommend cesarean delivery. However, to determine the optimal mode of delivery, additional comprehensive study with a large sample size is vital. If definitive management is warranted after delivery, we need to take a consideration of the status of prolapse, patient’s choice, and family size.

## Background

Pelvic organ prolapse (POP) is a common gynecologic condition. Pelvic organ prolapse is thought to be caused by a number of factors; however, none of them completely account for its pathophysiology. The two most significant risk factors that predispose women to pelvic organ prolapsed are vaginal delivery and aging [1, 2].

Pelvic organ prolapse complicating pregnancy is a rare event, which either exists before or has an onset during pregnancy. Pelvic organ prolapse in pregnancy can cause maternal and fetal complications. Some of the maternal complications include: preterm labor; urinary tract infection, acute urinary retention, and maternal death. Besides these, the fetal complications include: neurological complications, bone fracture due to dystocia at labor, pulmonary insufficiency, multi-organ failure due to preterm delivery, septicemia, and maybe fetal death [2, 4].

Due to possible risks to both mother and fetus as well as the fact that the long-term effects are unclear, surgical repair of pelvic organ prolapse during pregnancy is not routinely undertaken. Prolapse treatment options have changed from terminating pregnancies to using pessary and bed rest in the Trendelenburg position [2–4].

Management of pregnancy with preexisting pelvic organ prolapse is challenging. Physicians may encounter scenarios that pose management dilemmas during pregnancy and birth and after.

Here, we present the conservative management of preexisting pelvic organ prolapse in pregnancy accompanied by preterm premature rupture of membranes, leading to a successful cesarean birth with no negative effects on the mother or fetus.

## Case presentation

The patient was a 35-year old Ethiopian woman, gravida-V, para-IV, who did not remember her last normal menstrual period (LNMP) but claimed to be amenorrheic for the past 8 months. Her gestation from early first trimester ultrasound was 32 weeks and 1 day. She was sent to the emergency obstetrics/gynecology department after she was referred from a primary hospital as a case of preterm pregnancy, pelvic organ prolapse, and preterm premature rupture of membranes. She presented with a complaint of passage of clear liquor of 10 hours duration. She received three antenatal visits from a nearby medical facility. She began to complain about vaginal bulge about 2 years after her most recent delivery, and it got worse every day after that. She felt ashamed of it, so she didn't want to receive any treatment.

Vital signs were within the normal range, and a physical examination revealed no abnormalities in other systems. On abdominal examination, fundal height was 32 cm, the fetus was in longitudinal lie in cephalic presentation, fetal heart rate was 150 beats/minute, and the uterus was soft, without contractions.

**Fig. 1:**
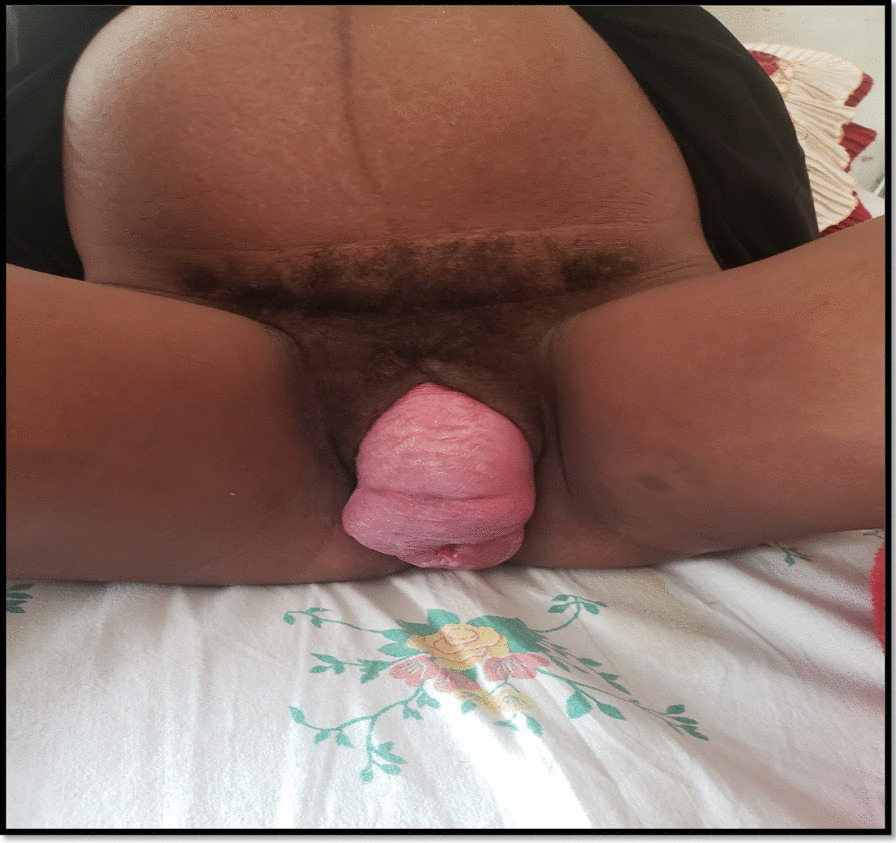
Stage IV pelvic organ prolapse

During a sterile Sims speculum examination, the entire uterine cervix and anterior vaginal wall was prolapsed and lying on the vulva, the external cervical orifice was closed with a minimal leakage of clear liquor. According to standard Pelvic Organ Prolapse Quantification (POP-Q IV) system, she was diagnosed with stage IV POP (Fig. 1, Table 1).

**Table 1:** A 3 × 3 grid system used for charting in the Pelvic Organ Prolapse Quantification (POP-Q) system regarding where six points are located with reference to the plane of the hymen in: two on the anterior vaginal wall (points Aa and Ba), two at the apical vagina (points C and D), and two on the posterior vaginal wall (points Ap and Bp). The genital hiatus (Gh), perineal body (Pb), and total vaginal length (TVL) are also measured. All POP-Q points, except TVL, are measured during patient's Valsalva maneuver and reflect maximum protrusion [11]

Aa:+3	Ba:+3	C:+8
Gh:4	Pb:4	Tvl:10
Ap:+3	Bp:+3	D:+7

On investigation, hemoglobin was 12.6 g/dl, white blood cell count was 9330/cm^3^, and urinalysis test was normal. Ultrasound revealed a singleton 32-week normal fetus and an amniotic fluid index (AFI) of 10 cm.

She was managed conservatively by manual reduction of prolapsed uterus and bed rest in the Trendelenberg position. Antenatal corticosteroids and prophylactic antibiotics were administered to accelerate fetal lung maturity and to prevent intraamniotic infection, respectively. Pessary was not inserted due to fear of infection. Then, she was admitted to the maternity ward for expectant management. In her expectant management follow-up, she did not have any complain except protruding mass per vagina during ambulation.

On her 37th week of pregnancy, a reevaluation of the prolapse was done, which revealed a stage 3 prolapse, the cervix noted to be 6 cm below the hymen. The patient gave birth to a healthy 3200 g male neonate with an Apgar score of 9−10 by elective cesarean section secondary to premature rupture of membrane at term. At the same operation, cesarean hysterectomy was done.

She was discharged on the fourth postpartum day. A postpartum follow-up at week 6 and month 6 showed that there was no pelvic prolapse when the patient was standing or walking. Follow-up is ongoing.

## Discussion

According to the few similar findings in the literature, pelvic organ prolapse during pregnancy is an incredibly rare condition [2–10]. Pregnancy-related surgical repairs of pelvic organ prolapse are only frequently carried out because the long-term effects are unknown, and there may be dangers to both the mother and the fetus.

Up until labor begins, conservative managements including manual cervix repositioning, vaginal pessary use, or pelvic floor exercises are advised [2–5]. Pessary, however, has the potential to worsen cervical edema, cause cervical ulceration, or both. Preterm birth or spontaneous abortion can result from a cervical infection. In previous study, maternal mortality from sepsis caused by an infected pessary was documented [2]. Furthermore, there is a lack of reported data in studies regarding the use of pessary to treat preexisting pelvic organ prolapse in pregnancies exacerbated by preterm premature membrane rupture. In our case, the pessary was not inserted because of fear of infection. Instead of using pessary treatments, we advise cautious care such as bed rest and manual uterine reduction.

Preexisting pelvic organ prolapse complicating preterm premature membrane rupture during delivery is difficult to manage since it presents multiple therapeutic conundrums. One study found that, despite its limited power, oxytocin during labor did not significantly enhance the chance of pelvic floor symptoms occurring, persisting, or worsening vaginal support in the early postpartum period [9].

Cervical dystocia, on the other hand, may occur if the proper cervical dilatation cannot be maintained because of prolapse. Obstructive labor, cervical laceration, and even rupture of the lower uterine segment can happen while labor is still in progress [2]. Women who arrive at or close to term with severe pelvic organ prolapse and preterm membrane rupture should have a cesarean delivery.

According to a case study, a 30-year-old multipara woman with stage 3 uterine prolapse was admitted to the hospital at 35 weeks of pregnancy while experiencing uterine contractions. The cesarean hysterectomy and sacrocolpopexy were carried out concurrently. They recommended cesarean hysterectomy as a treatment option, particularly for women who have finished having children and have significant pelvic organ prolapse [5].

A 33-year-old woman who was pregnant with twins and was referred to the clinic with labor contractions and complete uterine prolapse at 33 weeks of pregnancy was described in a similar case. If there was any sign of extreme fetal distress, an emergency cesarean section would be performed. In this study, abdominal hysteropexy employing rectus fascia strips was effectively carried out at the same procedure after a cesarean delivery [6].

Similarly, in a recent study, a 39-year-old woman underwent a caesarean section due to signs of a previous caesarean delivery and spontaneous membrane rupture at 37 weeks. Bilateral tubal ligation using Pomeroy’s approach was carried out at the same procedure after a cesarean delivery [3]. The difference is that, at 33 weeks of gestation, the entire uterine prolapse was resolved in this case, whereas it was not in our instance or either of the previous two case reports [5, 6]. We recommended cesarean hysterectomy, particularly for women who have finished having children and are experiencing severe pelvic organ prolapse.

## Conclusion

Managing women who already have pelvic organ prolapse can be challenging during pregnancy, delivery, and the postpartum period. Women with preexisting prolapse complicated by premature membrane rupture during the third trimester of pregnancy can be treated without application of a pessary. Our case shows the importance of conservative management, which includes strict antenatal follow-ups, lifestyle modifications, and manual uterine reduction. Due to potential intrapartum problems from induction of labor in the presence of severe pelvic organ prolapse, we recommend cesarean delivery. However, to determine the optimal the mode of delivery, more exhaustive research with a large sample size is essential. If definitive management is warranted after delivery, we need to take into consideration the status of prolapse, the patient’s choice, and their family size.

## Data Availability

The dataset used and/or analyzed during the current study are available from the corresponding author on reasonable request.

## References

[1] Swift S, Woodman P, O'Boyle A (2005). Pelvic organ support study (POSST): the distribution, clinical definition, and epidemiologic condition of pelvic organ support defects. Am J Obstet Gynecol.

[2] Tabaquero MA (2017). Pelvic organ prolapse in pregnancy. Obstet Gynecol Int J.

[3] Atılgan AE, Altuntaş SL (2020). Pregnancy with preexisting total uterine prolapse. Clin Med Rev Case Rep.

[4] De Vita D, Giordano S (2014). Two successful natural pregnancies in a patient with severe uterine prolapsed: a case report. J Med Case Rep.

[5] Meydanli MM, Ustun Y, Yalcin OT (2006). Pelvic organ prolapse in pregnancy complicating third trimester pregnancy. A case report. Gynecol Obstet Invest.

[6] Karatayli R, Gezgnic K, Kantarci AH, Car A (2013). Successful treatment of uterine prolapse by abdominal hysteropexy performed during cesarean section. Arch Gynecol Obstet.

[7] Martines-Verea A, Nohales-Alfonso F, Almela V, Peral A (2013). Arabian cerclage pessary as a treatment of an acute urinary retention in a pregnant woman with uterine prolapse. Case Rep Obstet Gynecol.

[8] Masumolto T, Nish M, Yokota M, Ito M (1999). Laparoscopic treatment of uterine prolapse during pregnancy. Obstet Gynecol.

[9] Nicola L, Yang J, Egger MJ, Nygaard IE (2021). Effect of oxytocin for induction and augmentation of labour on pelvic floor symptoms and support in postpartum period. Female Pelvic Med Reconstr Surg.

[10] Gupta R, Tickoo G (2012). Persistent uterine prolapsed during pregnancy and labour. J Obstet Gynecol India.

[11] Madhu C, Swift S, Moloney-Geany S, Drake MJ (2018). How to use the pelvic organ prolapse quantification (POP-Q) system?. Neurourol Urodyn.

